# Fluoride-related changes in the fetal cord blood proteome; a pilot study

**DOI:** 10.1186/s12940-024-01102-1

**Published:** 2024-07-23

**Authors:** Sami T. Tuomivaara, Susan J. Fisher, Steven C. Hall, Dana E. Goin, Aras N. Mattis, Pamela K. Den Besten

**Affiliations:** 1 Department of Obstetrics, Gynecology, and Reproductive Sciences Sandler-Moore Mass Spectrometry Core Facility, University of California, San Francisco, CA USA; 2 Department of Obstetrics, Gynecology, and Reproductive Sciences, Translational Research in Perinatal Biology and Medicine, University of California, San Francisco, CA USA; 3 Department of Obstetrics, Gynecology, and Reproductive Sciences, University of California, San Francisco, CA USA; 4 Program on Reproductive Health and the Environment, Department of Obstetrics, Gynecology, and Reproductive Sciences, University of California, San Francisco, CA USA; 5 Department of Pathology, University of Californa, San Francisco, CA USA; 6 Department of Orofacial Sciences, School of Dentistry, University of California, San Francisco, CA USA

## Abstract

**Background:**

Fluoride exposure during pregnancy has been associated with various effects on offspring, including changes in behavior and IQ. To provide clues to possible mechanisms by which fluoride may affect human fetal development, we completed proteomic analyses of cord blood serum collected from second-trimester pregnant women residing in northern California, USA.

**Objective:**

To identify changes in cord blood proteins associated with maternal serum fluoride concentration in pregnant women.

**Methods:**

The proteomes of 19 archived second-trimester cord blood samples from women living in northern California, USA, and having varied serum fluoride concentrations, were analyzed by quantitative mass spectrometry. The 327 proteins that were quantified were characterized by their abundance relative to maternal serum fluoride concentration, and subjected to pathway analyses using PANTHER and Ingenuity Pathway Analysis processes.

**Results:**

Pathway analyses showed significant increases in process related to reactive oxygen species and cellular oxidant detoxification, associated with increasing maternal serum fluoride concentrations. Pathways showing significant decreases included complement cascade, suggesting alterations in alterations in process associated with inflammation.

**Conclusion:**

Maternal fluoride exposure, as measured by serum fluoride concentrations in a small, but representative sample of women from northern California, USA, showed significant changes in the second trimester cord blood proteome relative to maternal serum fluoride concentration.

**Supplementary Information:**

The online version contains supplementary material available at 10.1186/s12940-024-01102-1.

## Introduction

Tooth enamel fluorosis, a biomarker for fluoride exposure, has steadily increased in the US [1, 2] resulting in increased risk for dental disease [3]. While the increase in fluorosis has drawn attention to concerns related to increased systemic toxicity of fluoride, recent measures of maternal serum, urine and amniotic fluid concentrations in pregnant women in northern California, are similar to concentrations measured in early water fluoridation studies in the 1950s [4]. This suggests that the increase in enamel fluorosis may be compounded by other, yet to be determined systemic factors.

However, studies of effects of maternal fluoride exposure on the developing fetus [5–10], including recent systematic reviews and meta-analyses, continue to raise questions related to the possibility of a dose-effect of fluoride on children’s neurodevelopment [11–13]. Rodent models also show fluoride-related effects on behavior and neurodevelopment [14, 15]. Mechansims for fluoride-related effects in rodent models include increased oxidative stress and inflammation [16–20]. However, the relevance of animal models to low level fluoride exposure in humans remains in question, as rodents require 5 to 10-fold higher levels of fluoride in drinking water to achieve plasma fluoride levels similar to those found in humans [21].

To investigate posssible effects of systemic fluoride exposure on fetal development in humans, we compared the cord blood proteomes from second trimester pregnant women residing in communities with various levels of water fluoridation in northern California. The placenta forms a barrier to macromolecules, including most proteins, to cross from maternal to fetal circulation [22], and therefore, cord blood proteins are primarily of fetal origin. Fluoride does cross the placenta [4, 23], and thus, changes in cord blood proteins relative to maternal serum fluoride, indicates an association between maternal fluoride ingestion and fetal developmental outcomes in humans.

## Methods

### Study samples

Cord blood samples were collected from 138 second trimester pregnant women with uncomplicated pregnancies from Northern California, seeking abortions at Zuckerberg San Francisco General Hospital. The study was approved by the University of California, San Francisco Committee on Human Research, and all women consented for sample collection. Following consent, maternal urine and serum were collected, and when possible, amniotic fluid and cord blood was also collected from each individual woman. Samples were labeled with a unique identification and barcode and then aliquoted into smaller Cryovials^®^ and stored at -80 ^o^C.

Samples from 48 individuals with urine, serum and amniotic fluid women were selected for fluoride analysis, with an equal number selected from collection dates in 2014, 2015, and 2016. All women resided in communities with water fluoride levels ranging from 0.2 to 0.9 ppm. The community water fluoride concentration recommended by CDC was 1.0 ppm prior to 2015, and was subsequently changed to 0.7 ppm fluoride in 2015, and therefore all were near or below the maxium recommended levels for artificial water fluoridation. Samples of maternal urine, serum, and amniotic fluid were analyzed for fluoride concentration using a fluoride ion selective electrode following acid diffusion, as previously described [4]. Our finding of significant positive associations between fluoride concentrations in maternal serum, urine, and amniotic fluid, and community water fluoride levels, indicate a stable residence of subjects who contributed samples, in their communities [4].

Cord blood samples were available from some of this cohort of 48 subjects, and we identified samples from 19 women, with the highest (*n* = 9) and lowest (*n* = 10) concentrations of maternal serum fluoride. Fluoride measurements in serum require at least 1 ml of sample, and given the limited amount of cord blood collected, it was not possible to use the cord blood samples both for fluoride concentration measurements and proteome analysis. Therefore, we used measurements of maternal serum fluoride concentrations as a biomarker for maternal fluoride exposure.

### Proteomics sample preparation and analysis

The serum samples were doped with protease inhibitors (100 µM AEBSF, 10 µM E-64, 10 µM Bestatin, 10 µM Pepstatin A, 5 mM EDTA), and cleared by centrifugation. The samples were subjected to Multiple Affinity Removal System (MARS-14, Agilent Technologies) that immunodepletes the 14 most abundant proteins, including serum albumin, from the samples. Total protein concentration of the depleted samples was determined with BCA protein assay. The samples were concentrated and buffer exchanged into 250 mM ammonium bicarbonate (doped with Bestatin and Pepstatin A) using 3000 MWCO spin filters (0.5 mL Amicon Ultra, Millipore Sigma), denatured with 7.5 M urea, reduced with 10 mM TCEP, alkylated with 40 mM iodoacetamide, diluted with 250 mM ammonium bicarbonate such that urea concentration was reduced to 1.25 M, and digested with trypsin (1:25 trypsin: total protein ratio). The resulting peptide mixtures were desalted using C18 SOLA SPE columns (ThermoFisher Scientific). Aliquots containing 1 µg of peptide material were analyzed in duplicate injections by liquid chromatography-tandem mass spectrometry (LC-MS/MS) on a system consisting of Digital PicoView nanospray source (New Objective) interfacing Eksigent 425 NanoLC pump and TripleTOF 6600 mass spectrometer (AB SCIEX). The mass spectrometry data was analyzed with ProteinPilot (v5.0.2, AB SCIEX) against non-fragment human proteins that have protein or RNA level evidence (45,481 canonical and isoform entries) retrieved from UniProt [24] Protein quantitation was performed with Skyline [25, 26] (v23.1) using MS1 filtering against spectral library prepared from the detected peptides with 95% or higher confidence. Peptide integration limits for quantitation were manually curated and at least two peptides were required for a protein quantitation. Two missed trypsin cleavages were allowed and carbamidomethyl on cysteine was set as static modification. Peptide intensities were normalized to the total ion current.

### Statistical analyses

Demographics were compared by the Kruskal-Wallis one-way analysis of variance test. For proteome analyses, batch effects were removed from the log_2_-transformed proteomics data with ComBat [27] function in the sva package (v3.52.0) using non-parametric location-scale mode without covariates in the R computing environment (http://www.r-project.org). Simple linear regression of protein abundances (non-log-transformed) against the maternal cord blood serum fluoride concentration without covariates was performed in R using the lm function. Multiple hypothesis testing correction for the detected proteins was performed using Benjamini-Hochberg procedure [28] in R using the p.adjust function. Pathway analyses were performed with Protein ANalysis THrough Evolutionary Relationships [29, 30] (PANTHER, release 18) against Gene Ontology (GO) Biological Process and Molecular Function annotations using statistical enrichment test without multiple testing correction, and QIAGEN Ingenuity Pathway Analysis [31] (IPA, v111725566). For both of these analyses, proteins were assigned a score by multiplying the inverse of the *p*-value and the sign of the slope parameter [32] from the linear regression analysis. Redundant pathways, as well as pathways with small number of detected proteins (< 6) or with *p*-value > 0.05 were filtered out, and the remaining pathways were ranked either by the *p*-value (PANTHER), or by the z-score (IPA).

## Results

Demographics of the study population showed community water fluoride levels at the time of sample collection from the 10 women with the lowest serum fluoride concentrations (0.0040 to 0.0073 ppm) ranged from 0.2 to 0.8 ppm. Community water levels at the time of sample collection, for 9 women with higher serum fluoride (0.0224 to 0.0593 ppm fluoride) ranged from 0.16 to 0.9 ppm. Community water fluoride concentrations for all subjects were near or below 1.0 ppm, which was the optimal water fluoride level recommended by the Center for Disease Control (CDC) at the time of collection.

The group of women with higher serum fluoride levels was further divided into middle range serum fluoride of 0.0224 to 0.0291 ppm fluoride, and high serum fluoride ranging from 0.0444 to 0.0593 ppm fluoride. There were no significant differences in maternal age, or ethnicity between the fluoride groups (Table 1). However, the higher community water fluoride levels were associated with higher serum fluoride concentrations, with significant differences between groups.

**Table 1:** Demographics of the study population (*N* = 19)

	Low serum fluoride	Middle serum fluoride	High serum fluoride	*p*-value
*N*	10	6	3	
Mean (SD)				
Serum fluoride (ppm)	0.006 (0.001)	0.025 (0.003)	0.052 (0.007)	0.00
Water fluoride (ppm)	0.36 (0.32)	0.59 (0.26)	0.80 (0.11)	0.05
Age	24.0 (3.9)	23.8 (3.5)	25.3 (4.6)	0.82
BMI	27.3 (5.8)	25.5 (3.4)	27.5 (3.5)	0.80
*N* (%)				
Race/ethnicity				
White	5 (50.0)	3 (50.0)	2 (66.7)	0.88
Black	3 (30.0)	2 (33.3)	1 (33.3)	0.99
Latina	2 (20.0)	1 (16.7)	0 (0.0)	0.72

### Proteomic analysis

**Fig. 1:**
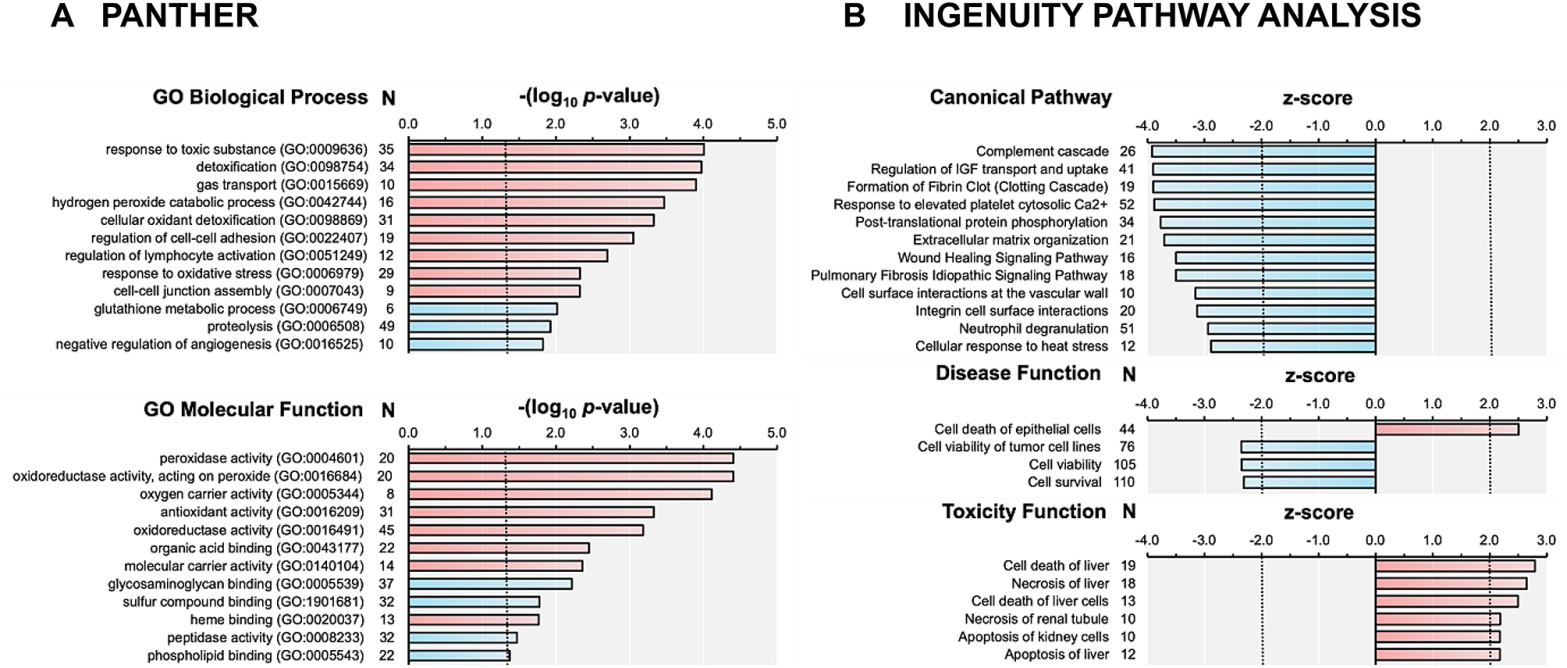
Pathway analyses of the quantitative proteomics data. **A**. Statistical enrichment test in PANTHER using Biological Process and Molecular Function Gene Ontology annotations. Up- and down-regulated pathways are respectively indicated by red and blue bars. Significance limits for -log10 (*p*-value) (> 1.3, corresponding to *p*-value < 0.05) are indicated by dashed lines, and twelve pathways with the highest *p*-value from the statistical enrichment test are shown. **B**. Ingenuity Pathway Analysis (IPA) using Canonical Pathway, and Disease and Toxicity annotations. Activated and inactivated pathways are respectively indicated by red and blue bars. Significance limits for the z-score (< -2.0 or > 2.0) are indicated by dashed lines. IPA calculates the z-score for a pathway as z = (N_+_ - N_−_) / √N, where N_+_ and N_−_ are respectively the numbers of (detected) up- and downregulated protein in the pathway, and N is the total number of detected proteins in the pathway. For the Canonical Pathways, twelve pathways with the highest z-score are shown, whereas all significant pathways are shown for the Disease and Toxicity Functions. For all analyses, N is number of proteins in the given pathway that were quantified by mass spectrometry

Altogether, our mass spectrometry analyses of cord blood sera quantified 327 proteins from the 19 serum samples. Simple linear regression of the detected proteins against the maternal blood fluoride concentration yielded several proteins that had statistically significant (*p*-value < 0.05) up- or down-regulation in related to increasing maternal fluoride levels (Appendix, Fig. 1). Upon Benjamini-Hochberg multiple test correction, however, none of these proteins retained significant *p*-value.

Nevertheless, the number of proteins identified allows the collective analysis of the dataset using pathway analysis approach. Statistical enrichment analysis using Gene Ontology (GO) Biological Process annotations showed elevated response to reactive oxygen species and oxidative stress generally. Concomitantly, proteins with GO Molecular Function annotations related to peroxidase, antioxidant, and other redox-related activities were increased (Fig. 1a, Appendix Table 1). Ingenuity Pathway Analysis (IPA) indicates that proteins associated with several physiological processes in blood, including complement and blood clotting cascade were down-regulated in response to increasing maternal fluoride concentration (Fig. 1b, Appendix Table 1). Additionally, the changes in protein abundance correlate to those observed in increased cellular death, decreased cell viability, and known toxicity profiles of liver and kidney (Fig. 1b, Appendix Table 1).

## Discussion

Serum fluoride concentrations represent a baseline burden of fluoride in balance between absorption and release from mineralizing tissue, and filtration and removal from the kidneys and liver. Maternal serum fluoride concentrations are therefore an accurate biological marker of fluoride exposure. Previous studies have shown that fetal cord blood fluoride concentrations reflect maternal blood fluoride [33], and therefore in this study we used maternal serum fluoride concentrations as a biomarker for fetal fluoride exposure [4].

Pathway analyses of cord blood proteins from 19 second trimester pregnant women from various communities across Northern California, with varying levels of serum fluoride concentrations, showed a significant associations between maternal serum fluoride and fetal cord blood proteins. In PANTHER, statistical enrichment analysis using Gene Ontology (GO), a user-provided ranked list of genes is statistically compared against discrete sets of genes (or gene products) with various GO annotations using Mann-Whitney U Test also known as Wilcoxon Rank-Sum Test [29, 30]. Most interesting were the results of the GO pathway analyses, which indicated that proteins whose abundances are significantly correlated with maternal fluoride concentrations were associated with oxidative stress and related biological pathways. The GO Biological Pathways showed upregulation in “response to toxic substances” and “cellular oxidant detoxification”. Similarly, GO Molecular Function analyses showed “upregulation of peroxidase activity processes”, and “oxidoreductase activity, acting on peroxide processes”. Peroxidases are a family of isoenzymes that oxidize reactive oxygen species [34], and cellular oxidative stress is known to occur when reactive oxygen species accumulate as a result of an imbalance between the production of reactive oxygen species and the activity of the cellular antioxidant defence mechanisms.

In Ingenuity Pathway Analysis, a user-provided ranked set of genes (or gene products) is statistically mapped on full Ingenuity Knowledge Base that contains manually curated and contextualized data from the published literature, and the subnetworks with densest matches to the user’s list are returned as the most significant [31]. This orthogonal information to Gene Ontology information, reveals that canonical pathways related to compliment cascade are down-regulated in response to increasing maternal fluoride concentration (Fig. 1b). Decreases in compliment cascade is known to be associated with inflammation [35]. It is noteworthy that, at the level of organs, IPA pathway analysis highlights the negative effects of increasing fluoride in the kidneys and liver function. Both of these organs play crucial roles in systemic detoxification, and are known to accumulate more fluoride than other organs [36]. Preterm cord blood contains high numbers of immature hematopoietic progenitors and endothelial/mesenchymal stem cells [37], and is perhaps the reason that the changes in proteins relative to maternal serum fluoride, were associated with pathways related to liver toxicity in the Ingenuity Pathway analyses.

Fluorine is one of the most reactive elements with high electronegativity and the second-highest electron affinity. While animal studies and cell culture studies have shown that fluoride ion affects oxygen metabolism to induce oxygen radicals enhancing oxidative stress at the cellular and molecular levels [38], this study is the first to identify a relationship between systemic fluoride concentrations and oxidative stress in humans. Oxidative stress is known to result in developmental neurotoxicity [39], and therefore is of critical importance in fetal development. Oxidative stress is also associated with increased inflammation [40], which also has negative effects on neurodevelopment [41].

In summary, our findings in this pilot study reveal that maternal plasma fluoride concentration is significantly associated with pathways related to oxidative stress, and provides the first human evidence of fluoride related mechanisms, previously identified in animal studies. The findings of this pilot study indicate the need for further studies on possible mechansims by which low level fluoride exposure during pregnancy may alter fetal development.

### Electronic supplementary material

Below is the link to the electronic supplementary material.

Supplementary Material 1

## Data Availability

No datasets were generated or analysed during the current study.
