# Supplementary material for: Fluoride-related changes in the fetal cord blood proteome; a pilot study
Source: Environ Health. 2024 Jul 23;23:66. doi: 10.1186/s12940-024-01102-1 (PMC11267808; doi:10.1186/s12940-024-01102-1)
Supplement: Supplementary file 1 — Supplementary Material 1 [file 12940_2024_1102_MOESM1_ESM.docx]

**Supplementary Data to**

Fluoride-related changes in the fetal cord blood proteome; a pilot study.

Sami T. Tuomivaara, Susan J. Fisher, Steven C. Hall, Dana E. Goin, Aras N. Mattis,

Pamela K. Den Besten

| **a** | |
| --- | --- |
|  | |
| **Hemoglobin Subunit Delta (HBD)**  *p* = 0.00889 | **Hemoglobin Subunit Epsilon 1 (HBE1)**  *p* = 0.02476 |
| 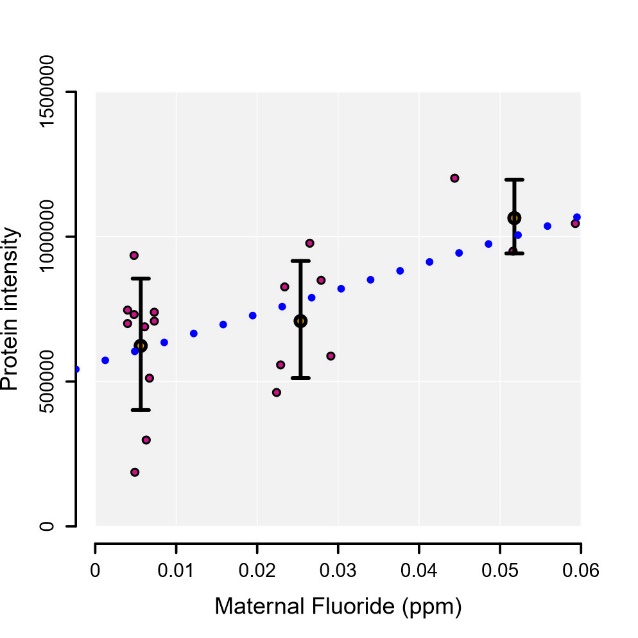 | 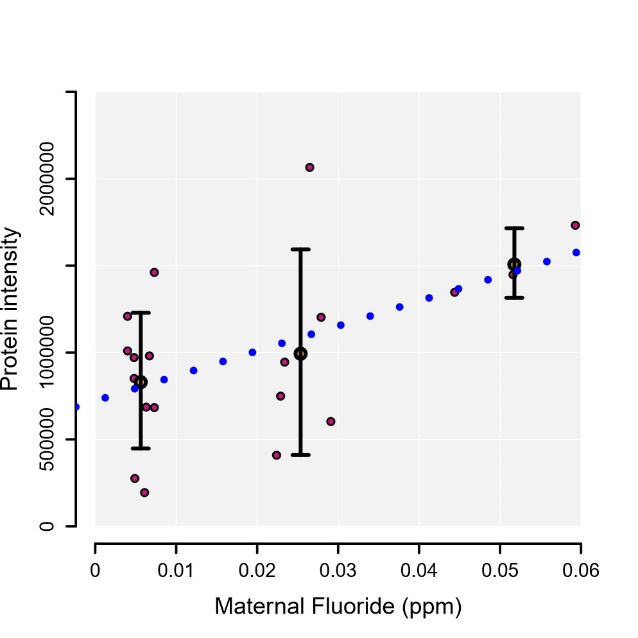 |
|  |  |
| **S100 Calcium Binding Protein A11 (S100A11)**  *p* = 0.03827 |  |
| 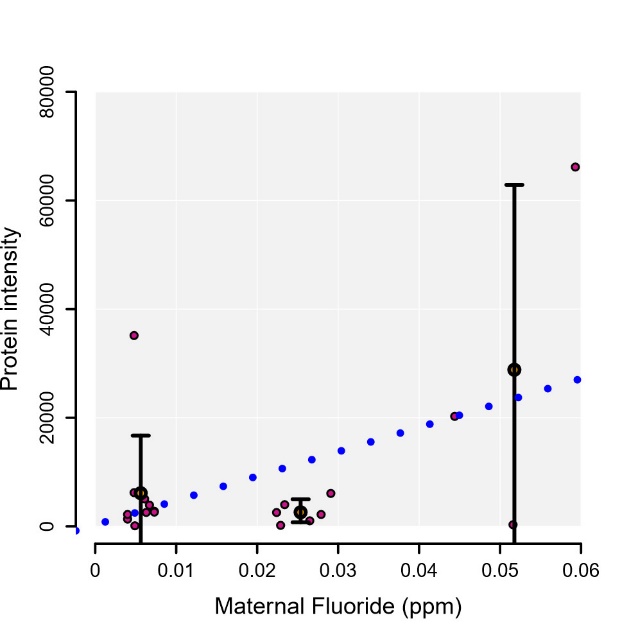 |  |
|  |  |
| **b** | |
|  | |
| **Complement Factor Properdin (CFP)**  *p* = 0.00311 | **Phosphoglucomutase 2 (PGM2)**  *p* = 0.00586 |
| 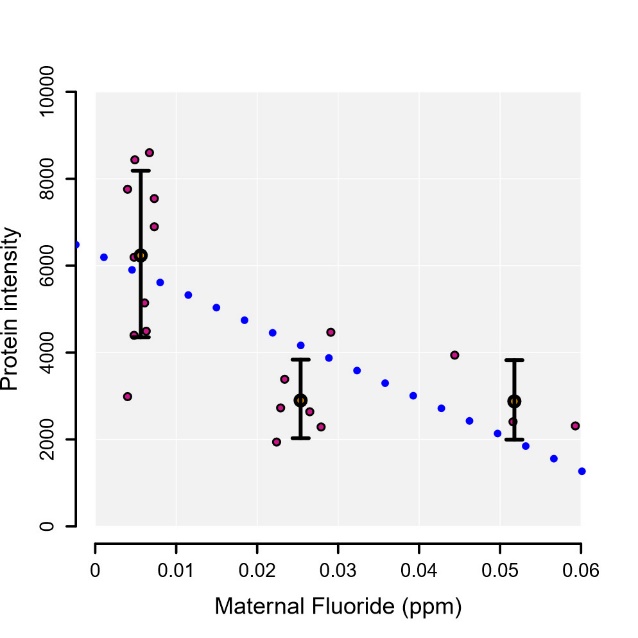 | 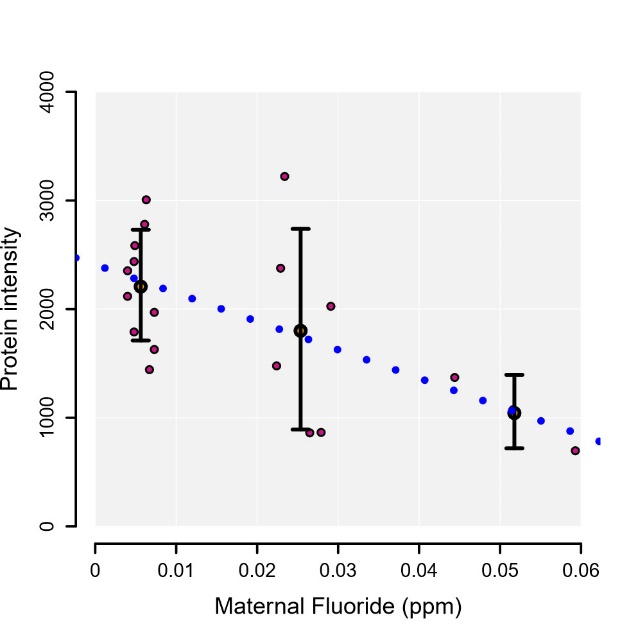 |
|  |  |
|  |  |
| **Small Proline-Rich Protein 1B (SPRR1B)**  *p* = 0.00822 | **Procollagen C-Endopeptidase Enhancer (PCOLCE)**  *p* = 0.01196 |
| 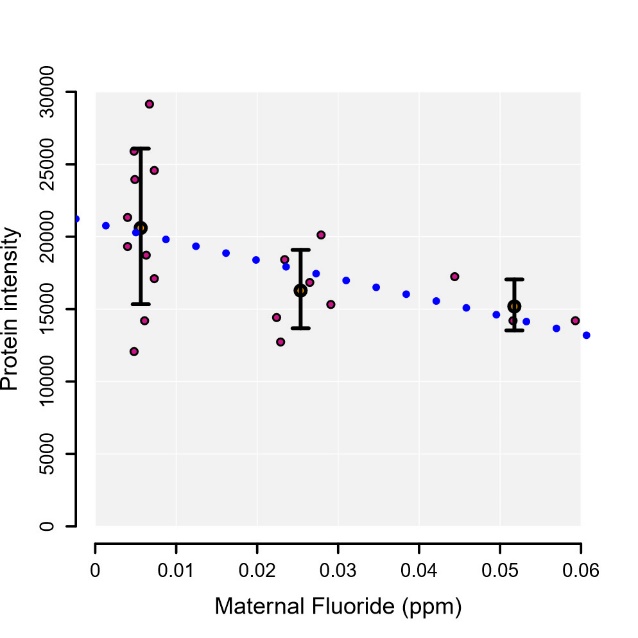 | 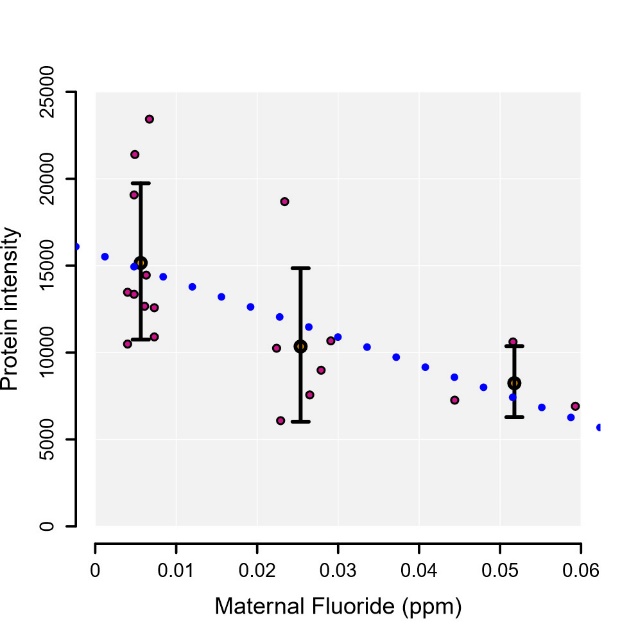 |
|  |  |
|  |  |
|  |  |
|  |  |
|  |  |
| **Prostaglandin D2 Synthase (PTGDS)**  *p* = 0.02003 | **Pyruvate kinase (PKM)**  *p* = 0.02466 |
| 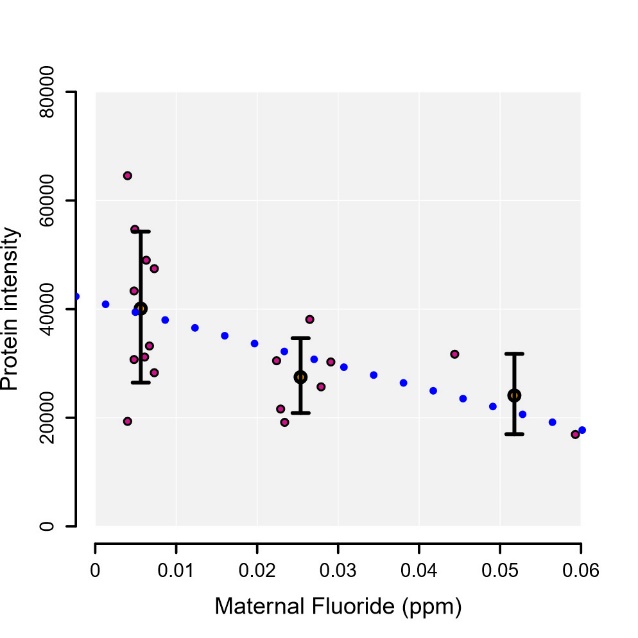 | 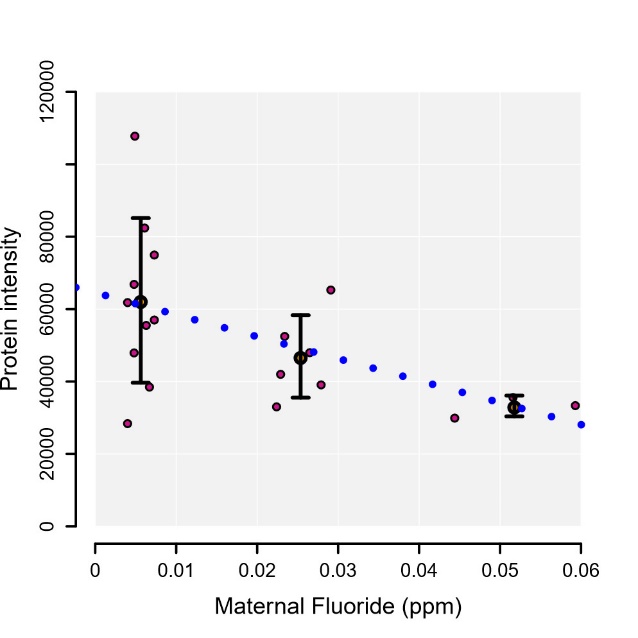 |
|  |  |
| **Meprin A Subunit Alpha (MEP1A)**  *p* = 0.02627 | **Galectin 3 Binding Protein (LGALS3BP)**  *p* = 0.02651 |
| 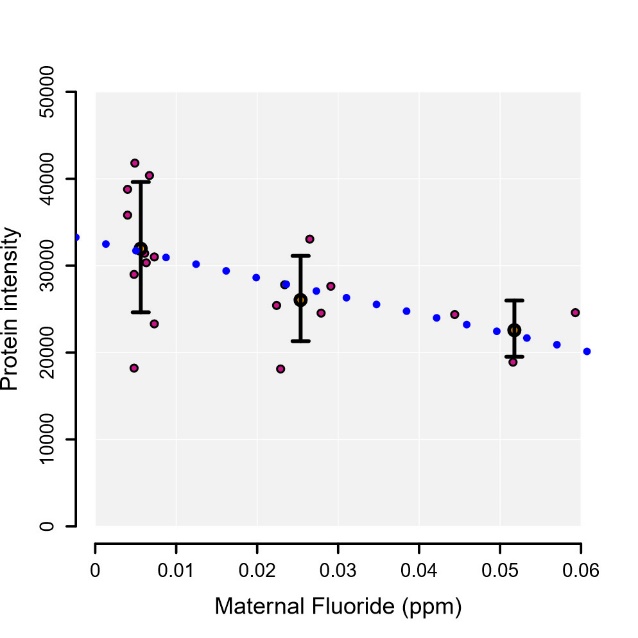 | 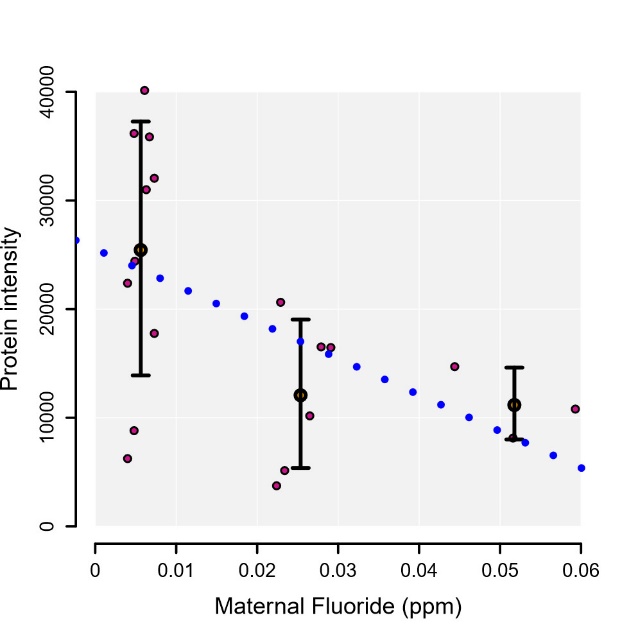 |
|  |  |
|  |  |
|  |  |
|  |  |
|  |  |
| **Prothrombin (F2)**  *p* = 0.02719 | **Hydroxysteroid (17-beta) dehydrogenase 1 (HSD17B1)**  *p* = 0.04137 |
| 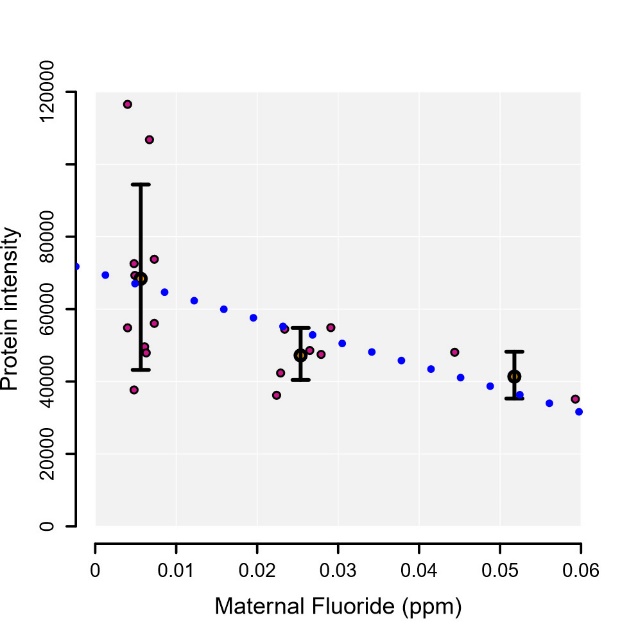 | 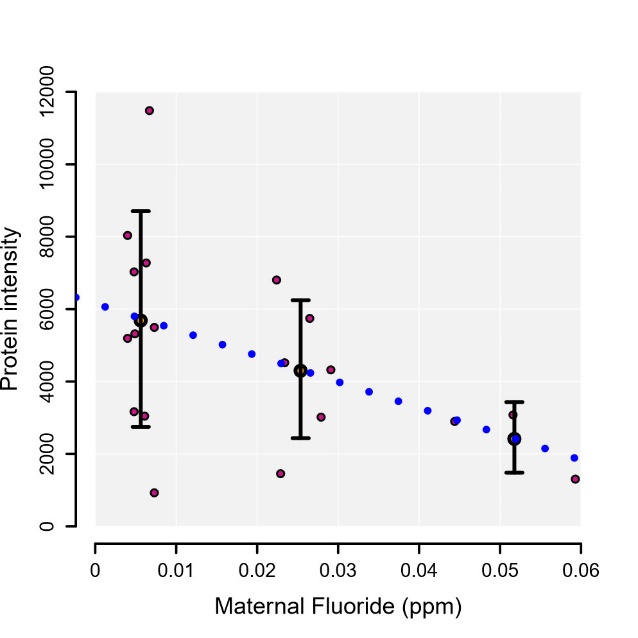 |
|  |  |
| **Tubulin Polymerization Promoting Protein Family Member 3 (TPPP3)**  *p* = 0.04263 | **Complement C1r subcomponent (C1R)**  *p* = 0.04553 |
| 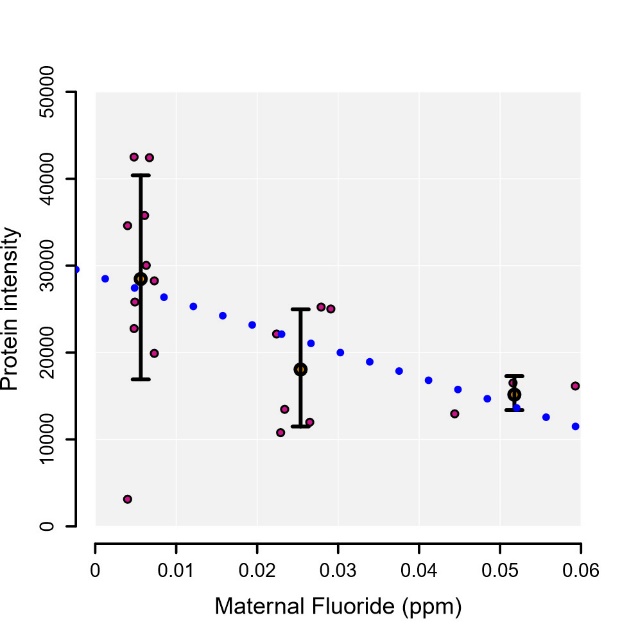 | 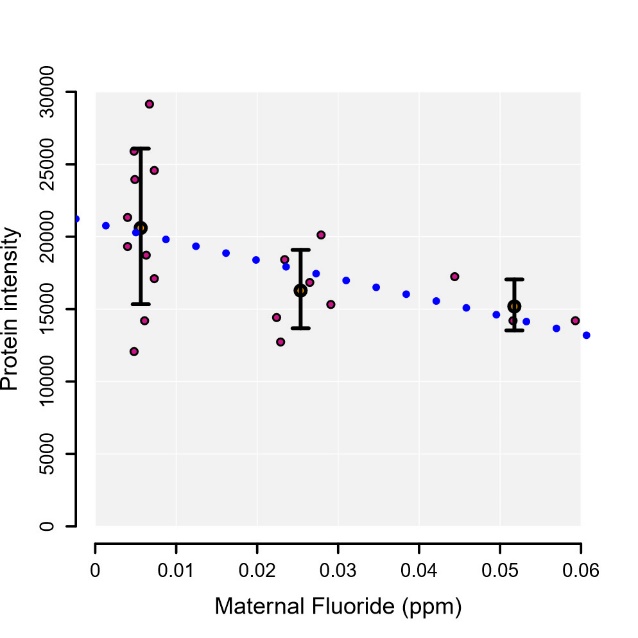 |
|  |  |
|  |  |
| **Supplementary Figure 1.** Protein intensity data v. maternal fluoride concentration for proteins whose linear regression analysis slope *p*-value is < 0.05. *p*-value for the slope (below the protein name), as well as the linear regression line (in blue) are indicated. **a** Proteins whose abundance increases in increasing maternal fluoride concentrations. **b** Proteins whose abundance decreases in increasing maternal fluoride concentrations. Mean ± standard deviation of the protein intensities are indicated for the low [0.0040 – 0.0073 ppm], middle [0.0224 – 0.0291 ppm], and high [0.0444 – 0.0593 ppm] groups. | |
|  |  |

**Supplementary Table 1.** Results from PANTHER and Ingenuity Pathway Analysis (IPA). N = number of proteins in the pathway that were detected by mass spectrometry. + = upregulation (for PANTHER) or activation (for IPA), - = downregulation (for PANTHER) or deactivation (for IPA).

| **PANTHER – GO Biological Process** | | | | | |
| --- | --- | --- | --- | --- | --- |
| **Pathway** | **GO term** | **N** | **+/-** | ***p*-value** | **-log10**  **(*p-*value)** |
| response to toxic substance  detoxification  gas transport  hydrogen peroxide catabolic process  cellular oxidant detoxification  regulation of cell-cell adhesion  regulation of lymphocyte activation  response to oxidative stress  cell-cell junction assembly  glutathione metabolic process  proteolysis  negative regulation of angiogenesis | GO:0009636  GO:0098754  GO:0015669  GO:0042744  GO:0098869  GO:0022407  GO:0051249  GO:0006979  GO:0007043  GO:0006749  GO:0006508  GO:0016525 | 35  34  10  16  31  19  12  29  9  6  49  10 | +  +  +  +  +  +  +  +  +  +  -  - | 0.00010  0.00011  0.00013  0.00034  0.00047  0.00089  0.00200  0.00472  0.00475  0.00968  0.01190  0.01500 | 4.00877  3.97469  3.90309  3.47108  3.32790  3.04915  2.69897  2.32606  2.32331  2.01412  1.92445  1.82391 |
|  |  |  |  |  |  |
| **PANTHER – GO Molecular Function** | | | | | |
| **Pathway** | **GO term** | **N** | **+/-** | ***p*-value** | **-log10**  **(*p*-value)** |
| peroxidase activity  oxidoreductase activity, acting on peroxide  oxygen carrier activity  antioxidant activity  oxidoreductase activity  organic acid binding  molecular carrier activity  glycosaminoglycan binding  sulfur compound binding  heme binding  peptidase activity  phospholipid binding | GO:0004601  GO:0016684  GO:0005344  GO:0016209  GO:0016491  GO:0043177  GO:0140104  GO:0005539  GO:1901681  GO:0020037  GO:0008233  GO:0005543 | 20  20  8  31  45  22  14  37  32  13  32  22 | +  +  +  +  +  +  +  -  -  +  -  - | 0.00004  0.00004  0.00008  0.00047  0.00066  0.00357  0.00436  0.00611  0.01670  0.01720  0.03370  0.04270 | 4.40894  4.40894  4.11182  3.32790  3.18111  2.44733  2.36051  2.21396  1.77728  1.76447  1.47237  1.36957 |

|  |  |  |  |  |
| --- | --- | --- | --- | --- |
| **Ingenuity Pathway Analysis – Canonical Pathways** | | | | |
| **Pathway** | **N** | **+/-** | **z-score** | **p-value** |
| Complement cascade  Regulation of IGF transport and uptake  Formation of Fibrin Clot (Clotting Cascade)  Response to elevated platelet cytosolic Ca2+  Post-translational protein phosphorylation  Extracellular matrix organization  Wound Healing Signaling Pathway  Pulmonary Fibrosis Idiopathic Signaling Pathway  Cell surface interactions at the vascular wall  Integrin cell surface interactions  Neutrophil degranulation  Cellular response to heat stress | 26  41  19  52  34  21  16  18  10  20  51  12 | -  -  -  -  -  -  -  -  -  -  -  - | -3.922  -3.904  -3.900  -3.883  -3.773  -3.710  -3.500  -3.500  -3.162  -3.130  -2.941  -2.887 | 2.51E-23  3.16E-47  2.51E-25  5.01E-63  1.26E-38  2.51E-19  1.15E-07  1.26E-07  4.17E-04  2.51E-20  6.31E-33  6.17E-09 |
|  |  |  |  |  |
| **Ingenuity Pathway Analysis – Disease Functions** | | | | |
| **Pathway** | **N** | **+/-** | **z-score** | ***p*-value** |
| Cell death of epithelial cells  Cell viability of tumor cell lines  Cell viability  Cell survival | 44  76  105  110 | +  -  -  - | 2.505  -2.355  -2.350  -2.312 | 3.34E-13  6.21E-19  5.67E-24  2.06E-25 |
|  | | | | |
| **Ingenuity Pathway Analysis – Toxicity Functions** | | | | |
| **Pathway** | **N** | **+/-** | **z-score** | ***p*-value** |
| Cell death of liver  Necrosis of liver  Cell death of liver cells  Necrosis of renal tubule  Apoptosis of kidney cells  Apoptosis of liver | 19  18  13  10  10  12 | +  +  +  +  +  + | 2.795  2.650  2.498  2.184  2.181  2.177 | 2.11E-06  6.01E-06  4.74E-04  6.43E-07  3.13E-05  8.69E-05 |
